# Efficacy of Cyanoacrylate Glue Ablation of Primary Truncal Varicose Veins Compared to Existing Endovenous Techniques: A Systematic Review of the Literature

**DOI:** 10.1055/s-0040-1708866

**Published:** 2020-06-19

**Authors:** Anthony Pio Dimech, Kevin Cassar

**Affiliations:** 1 Vascular Unit, Department of Surgery, Mater Dei Hospital, Msida, Malta; 2 Vascular Unit, Department of Surgery, Mater Dei Hospital, Msida, Malta

**Keywords:** *n* -butyl 2 cyanoacrylate, radiofrequency, laser, saphenous vein, varicose veins

## Abstract

**Introduction** One-third of adults in the United States and United Kingdom suffer from varicose veins. *n* -butyl-2-cyanoacrylate (NBCA) glue is a novel endovascular, nontumescent, nonthermal ablation technique for treatment of this condition. It has proved effective in multiple studies since its first use in 2013. The aim of this systematic review is to assess the efficacy of NBCA in ablating primary truncal varicose veins and eliminating reflux compared with existing endovascular techniques. Secondary outcomes include complications and quality of life.

**Methods** PRISMA was used as a guide and studies were screened for risk of bias and methodological quality. Subjects had to be ≥18 years of age and followed-up posttreatment with color Duplex ultrasound (DUS). Eligibility criteria included saphenofemoral junction (SFJ) or saphenopopliteal junction (SPJ) incompetence with reflux down truncal veins lasting >0.5 seconds on DUS interrogation and a Clinical, Etiological, Anatomical, and Pathophysiological classification of venous disorders ranging between C1 and C6.

**Results** Out of 2,910 patients (3,220 veins) in 17 studies, 1,981 were administered NBCA, 445 radiofrequency ablation (RFA), and 484 endovenous laser ablation (EVLA) with mean procedure times of 25.7, 23.2, and 28.7 minutes, respectively. Mean recruitment period was 9 months (1–36 months) and followed-up for an average of 12.3 months (1–36 months). The majority were C2 to C3. Two-year occlusion rates were 93.7, 90.9, and 91.5% for NBCA, RFA, and EVLA, respectively. NBCA-treated patients experienced the least complications, with bruising, phlebitis, and pain being the most prevalent. Quality of life improved equally in all three modalities.

**Conclusion** NBCA is simple to administer, safe, and effective even without compression stockings. Further studies are required to assess longer-term benefit and the effect of anticoagulation on vein obliteration.

Thirty-five percent of adults in the United States and United Kingdom have chronic lower limb superficial venous disease. 1 Varicose veins are more common in females, with a predilection toward the older age group and may run in families. A body mass index >30 kg/m ^2^ is a risk factor for chronic venous insufficiency. 2 Symptoms include limb heaviness, ache, and edema. Skin changes such as spider veins, varicose veins, hemosiderin deposition, inflammation, lipodermatosclerosis, and ulceration often follow in untreated cases. 2 3 4

The 2013 National Institute for Health and Care Excellence (NICE) guideline on diagnosis and management of varicose veins (updated March 2018) recommends radiofrequency ablation (RFA) or endovenous laser ablation (EVLA) as first line treatment for truncal reflux. Second-line is ultrasound-guided foam sclerotherapy. Open surgery is indicated only if the other methods are unsuitable. Any incompetent tributaries are preferentially treated in the same session. Compression hosiery should not be used longer than 7 days after intervention, and is first choice only in pregnancy or if the previously mentioned interventions are unsuitable. 5 NICE also issued a specific guideline in 2015 on the use of *n* -butyl-2-cyanoacrylate (NBCA) for varicose veins but did not promote its routine use. 6 Almeida et al reported the first human application of NBCA for incompetent great saphenous veins (GSVs) in 2013. All 38 veins under study were obliterated at 48 hours and 92% at 1 year with minor short-lasting adverse effects. 7

The aim of this systematic review is to assess the efficacy of NBCA in ablating primary truncal varicose veins and eliminating reflux compared with existing endovascular techniques in the immediate, medium, and long-term settings. Secondary outcomes include complications, patient acceptability, and quality of life.

## Methods

### Protocol and Search Strategy

This review is registered in PROSPERO database (registration code: CRD42018106323) and followed the PRISMA checklist. 8 9 One author performed a literature search and data extraction up to October 2018 with no set date range and using established MeSH vocabulary in PubMed, EMBASE, Scopus, Cochrane Library, and ScienceDirect. Search terms were: “varicose vein,” “saphenous vein,” “glue,” “ *n* -butyl cyanoacrylate,” and “ *n* -butyl 2 cyanoacrylate.” References and article suggestions by search engines were assessed to identify more relevant studies. Duplicates were removed and further exclusions performed after reviewing abstracts. The chosen manuscripts were then scrutinized while applying inclusion and exclusion criteria.

### Inclusion and Exclusion Criteria

Human randomized controlled trials (RCTs), cohort studies, and case reports in English language involving the use of NBCA to treat primary truncal varicose veins (i.e., GSV, small saphenous vein [SSV], and anterior accessory saphenous vein [AASV]) were included. If more than one modality was used, the said manuscript was only included if the data for NBCA could be fully extracted. Studies excluding NBCA glue or comparing NBCA with treatments other than RFA, EVLA, or foam sclerotherapy were excluded. 1 2 10 11 Supplementary Table 1 (online only) summarizes patient characteristics for inclusion/exclusion.

### Primary and Secondary Outcomes

Primary outcome was successful obliteration of lumen of target vein, defined as occlusion of the entire treated vein segment with no discrete segments of patency exceeding 5 cm, confirmed on color Duplex ultrasound (DUS) after the procedure. 1 Follow-up DUS assessments at 3 days, 7 days, 1 month, 3 months, 6 months, 1 year, and 2 years were examined.

Influence of vein length, diameter, NBCA device, and postoperative compression stockings on early (3 months) and intermediate term (6 months, 1 year) occlusion rate was taken as secondary outcomes. Vein length was taken as a mean value incorporating GSVs, SSVs, and AASVs with no distinction between the three. Where a particular vein diameter was taken at different levels, the mean of these values was calculated.

Clinical, Etiological, Anatomical, and Pathophysiological classification and Varicose Clinical Severity Score (VCSS) were used to measure severity of varicose veins at baseline and postintervention. Quality of life was primarily investigated using the Aberdeen Varicose Vein Questionnaire (AVVQ). 2 “Thrombophlebitis” and “abnormal skin reactions” in treatment zones were included with the general term “phlebitis.” 12 13 All thrombus extensions into the deep venous systems were classified as deep vein thromboses (DVTs). Complications common to the three ablation modalities were evaluated.

### Data Extraction

Any uncertainties in the literature were discussed with the second author and the authors of the original manuscripts where applicable. Risk of methodological bias was explored using the Cochrane Risk of Bias tool for RCTs. 14 15 Quality assessment was performed using the Downs and Black quality assessment tool (for RCTs) and the National Heart, Lung and Blood Institute: Quality Assessment Tool for Before-After (Pre–Post) Studies With No Control Group (NHLBI-QAT). 16 17

### Statistical Analysis

Continuous variables were represented by means, standard deviations, and ranges. Categorical variables were shown in actual numbers and percentages. Scatter plots were created using Python version 3.7 (Python Software Foundation, Beaverton, DE). Statistical analysis was done using IBM SPSS Statistics software (IBM Corp. Released 2013. IBM SPSS Statistics for Windows, Version 22.0. Armonk, NY). Spearman's correlation and Mann-Whitney U-test were performed on groups of subjects at 3, 6, and 12-month intervals following NBCA treatment. These tests were chosen because continuous variables were not normally distributed. Level of statistical significance was taken as *p* < 0.05.

## Results

### Description of Studies

**Fig. 1:**
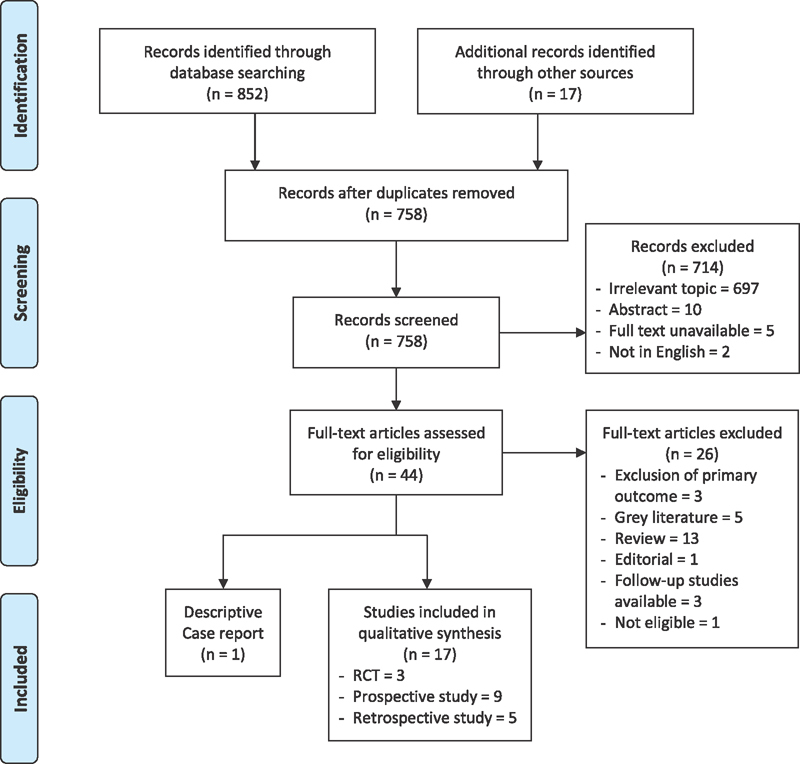
PRISMA flowchart depicting the process of selection of articles. RCT, randomized controlled trial.

The PRISMA flowchart ( Fig. 1 ) depicts the choice of manuscripts at different phases. One case report was identified but not reviewed as it contained heterogeneous data. 18 All were published in peer-reviewed indexed scientific journals. There were 3038 participants (3,220 veins). A subgroup of 128 patients were excluded because of the missing data. 19 20 21 Of the 2910 patients who were included, 1981 received NBCA, 445 RFA, and 484 EVLA. Comparison of NBCA with RFA and/or EVLA was performed in three RCTs and two retrospective studies. 10 12 19 21 22 No studies compared NBCA with sclerotherapy, but this was frequently used an adjunctive treatment. Levels of evidence for therapeutic studies were judged using criteria from the Centre for Evidence-Based Medicine. 23

### Quality and Risk of Bias Assessment

#### Randomized Controlled Trials

Risk of bias for RCTs is illustrated in Table 1 . Bozkurt and Yilmaz pseudorandomized their patients to alternate EVLA and NBCA. This led to a high risk of selection bias. 10 Randomization was better in the VeClose trial and the study by Eroglu and Yasim. 1 12 19 The former also included “roll-in cases” so that investigators could achieve familiarity with the NBCA procedure. DUS assessments were not always performed by blinded personnel. Attrition bias was unclear in two RCTs as drop-outs were not formally analyzed. 13 20 Effect of adjunctive therapies and postoperative compression stockings was not evaluated. Only one performed power analysis. 19 Primary and secondary end points were clearly reported in all RCTs.

**Table 1:** Traffic light plot illustrating risk of bias of the included RCTs (using the Cochrane risk of bias tool) and Downs and Black quality assessment scores

Abbreviation: RCT, randomized controlled trials.

Note: The score for item 27 in the Downs and Black checklist was modified to determine whether power analysis was conducted (yes = 1 point) or not (no = 0 points). So, the maximum score for the checklist was 28 instead of 32. 24

#### Prospective and Retrospective Studies

Prospective studies were of a higher methodological quality ( Supplementary Fig. 1 and Supplementary Table 2 [online only]). Selection bias toward bilateral varicose veins was observed in one prospective and one retrospective study. 25 26 Another reported a modification of intervention after commencement of data collection which improved the complication rate in the remaining patients. 27 Blinding of assessors was not possible. The loss to follow-up for NBCA was 23.7% in one manuscript. 28 Another started with 34 patients and had 26% loss at 1 month. 13 One prospective and one retrospective study reported percentage occlusion rate only once at 1 month and 1 year respectively despite mentioning several follow-up intervals in the methodology. 13 22 Coincidentally, the former did not have sufficient patients at the target 3-month interval to formulate strong conclusions. 13 Another study did not differentiate between the short- (1 week) and mid-term (2 months) outcome results, which instead were displayed as combined absolute values. 21

### Population and Operative Details

Study characteristics are summarized in Tables 2 and 3 .

**Table 2:** Characteristics of identified studies

First author	Year	Country	Study design, Evidence level	Single/Multicenter	Comparator	Recruitment period	NBCA patients	Comparator patients	Patients excluded	Proposed follow-up (mo)	Actual follow-up (mo)	Definition of varicose vein and/or vein incompetence
Bozkurt and Yilmaz 10	2016	Turkey	RCT, 1B	Multicenter	EVLA	December 2013–March 2014 (3 mo)	154	156 EVLA	–	12	12	CEAP C2-C4b with SFJ incompetence and GSV reflux lasting >0.5 s on DUS.
Morrison et al 12	2017	United States	RCT, 1B	Multicenter VeClose Trial	RFA	March–September 2013 (6 mo)	108	114 RFA	–	12	12	GSV reflux ≥0.5 s on DUS in the standing position.
Eroglu and Yasim 19	2018	Turkey	RCT, 1B	–	RFA, EVLA	November 2014–June 2015 (7 mo)	168	139 EVLA 149 RFA	69	24	24	GSV >5.5 mm and SSV >4 mm in diameter 2 cm below the SFJ and SPJ with the patient standing, and reflux >0.5 s.
Proebstle et al 33	2015	Europe (multinational)	Prospective, 2B	Multicenter	None	December 2011–July 2012 (7 mo)	70	–	–	12	12	Primary GSV incompetence diagnosed clinically +/− visible varicosities and confirmed by DUS. GSV diameter ≥3 mm and ≤10 mm on standing DUS.
Kolluri et al 34	2016	United States	Prospective, 2B	Multicenter	None	March–September 2013 (6 mo)	20	–	–	12	12	Moderate to severe varicosities and venous reflux in the GSV >0.5 s.
Çalık et al 29	2016	Turkey	Prospective, 2B	Multicenter	None	April–September 2014 (5 mo)	181	–	–	6	7.5	GSV insufficiency with >0.5 s of reflux.
Tekin et al 30	2016	Turkey	Prospective, 2B	Single center	None	January–July 2014 (6 mo)	62	–	–	6	8	Symptomatic incompetent GSV with a diameter of >5.5 mm, with or without visible varicosities.
Chan et al 25	2017	China	Prospective, 2B	Single center	None	September 2014–October 2015 (13 mo)	29	–	–	12	9	Retrograde SFJ flow ≥0.5 s on DUS with patient standing.
Gibson and Ferris 32	2017	United States	Prospective, 2B	Single center WAVES trial	None	October–December 2015 (3 mo)	50	–	–	1	1	Reflux of >0.5 s of retrograde flow in a varicose vein in the standing position.
Almeida et al 28	2017	Dominican Republic	Prospective, 2B	Single center	None	December 2010 (1 mo)	38	–	–	36	36	Clinical venous reflux disease in the GSV +/− varicosities, and confirmed by DUS.
Eroglu et al 20	2017	Turkey	Prospective, 2B	Single center	None	May–October 2014 (5 mo)	168	–	12	30	30	GSV diameter >5.5 mm and a SSV diameter >4 mm in conjunction with reflux >0.5 s.
Park 13	2017	South Korea	Prospective, 2B	Single center	None	December 2016–February 2017 (2 mo)	34	–	–	3	3	Saphenous vein with ≥0.5 s of reflux in the standing position with a diameter of at least 3 mm.
Koramaz et al 22	2017	Turkey	Retrospective, 2C	Single center	EVLA	May 2013–August 2014 (15 mo)	150	189 EVLA	–	12	12	GSV diameter ≥5.5 mm and ≤15 mm with reflux >0.5 s.
Chan et al 26	2017	China	Retrospective, 2C	Single center	None	September 2014–June 2016 (21 mo)	55	–	–	12	5	Retrograde flow of >0.5 s on DUS over the SFJ on standing.
Bademci et al 31	2018	Turkey	Retrospective, 2C	Single center	None	September 2015–September 2016 (12 mo)	50	–	–	12	12	GSV diameter of 5.5–10 mm with reflux >0.5 s.
Yavuz et al 27	2018	Turkey	Retrospective, 2C	Single center	None	April–July 2016 (3 mo)	538	–	–	12	12	GSV diameter at SFJ of ≥5.5 mm and ≤15 mm on standing. GSV reflux ≥0.5 s on DUS.
Yang et al 21	2019	Canada	Retrospective, 2C	Single center	RFA	January 2014–December 2016 (3 y)	106	182 RFA	47	2	2	Not defined.
Lane et al 18	2013	United Kingdom	Case report, 4	Single center	None	March 2012	1	–	–	6	6	Not defined.

Abbreviations: AASV, anterior accessory saphenous vein; CEAP, Clinical, Etiological, Anatomical, and Pathophysiological; DUS, duplex ultrasound; EVLA, endovenous laser ablation; GSV, great saphenous vein; NBCA, *n* -butyl-2-cyanoacrylate; RCT, randomized controlled trial; RFA, radiofrequency ablation; s, seconds; SFJ, saphenofemoral junction; SSV, small saphenous vein.

**Table 3:** Intraoperative characteristics of selected studies

Author	Ablation device	GSV count	SSV count	AASV count	Delivery catheter position distal to SFJ/SPJ (cm)	Volume of glue used mean ± SD (range) (mL)	Mean Vein diameter mean ± SD (range) (mm)	Treated segment length mean ± SD (range) (cm)	Procedure duration mean ± SD (range) (minutes)	Concomitant treatment of tributaries (e.g., foam, phlebectomy)	Concomitant GSV and SSV NBCA treatment	Concomitant treatment of GSV and SSV with other endovascular modality (e.g., foam)	Postoperative compression stockings
Bozkurt and Yilmaz 10	VariClose	154	0	0	3	–	7.2 ± 1.8	29.8 ± 5.4	15 ± 2.5	No	No	No	No
	Evlas	156	0	0	1.5	N/A	7.1 ± 1.6	29.7 ± 8.1	33.2 ± 5.7	No	No	No	Yes × 10 d
Morrison et al 12	VenaSeal	108	0	0	5	1.2 (0.4–2.3)	5.6	32.8 (8–61)	24 (11–40)	No	No	No	Yes, for 3 d continuously, then 4 more days during waking hours.
	ClosureFast	114	0	0	–	N/A	5.85	35.1 (6.5–84.5)	19 (5–46)	No	No	No	
Eroglu and Yasim 19	VariClose	159	9	0	3	–	7.6 ± 1.9	26.4 ± 6.5	15.3 ± 2.6	No	Yes	No	Yes, elastic compression bandage for 2 d, then Class 1 for 1 mo.
	ClosureFast	146	3	0	–	N/A	7.8 ± 1.9	27.6 ± 5.3	27.3 ± 7.7	No	Yes	No	
	Evlas	123	16	0	–	N/A	8 ± 1.9	27.1 ± 5.8	35.0 ± 5.2	No	Yes	No	
Proebstle et al 33	VenaSeal	70	0	0	5	–	7.8 ± 2.1 (6.6–14)	37.6 (7–72)	18.6 (8–74)	No	No	Yes	No
Kolluri et al 34	VenaSeal	20	0	0	5	1.1 (0.6–2.2)	6.1	31.4 (18–50)	31 (23–46)	–	No	No	–
Çalık et al 29	VariClose	206	9	0	3	0.9 (0.7–2.1)	5.85	31.6 ± 6.1 (23–70)	5.4 ± 2.5 (3–14)	Yes	Yes	Yes	Elastic bandages x1 d, no compression stockings.
Tekin et al 30	VariClose	62	0	0	5	1.5	7.5 ± 1.5 (5.5–13)	28 (20–40)	17 (9–37)	No	No	No	Elastic bandages x1 d, no compression stockings.
Chan et al 25	VenaSeal	57	0	0	4	–	7.1 (3.9–11.4)	27 (17–33)	64 (28–99)	Yes	No	No	Yes
Gibson and Ferris 32	VenaSeal	48	8	14	5	0.93 ± 0.3	7.7	24 ± 12.8	27 ± 11 (11–55)	No	Yes	No	No
Almeida et al 28	VenaSeal	38	0	0	3.5	1.3 (0.63–2.25)	–	33.2 ± 9.1	20 (11–33)	–	No	–	No
Eroglu et al 20	VariClose	159	9	0	3	2	7.4 ± 2.3 (5.5–14)	26.3 ± 6.5 (9–43)	15.3 ± 2.5 (10–25)	–	No	No	–
Park 13	VenaSeal	47	16	0	5	1.2 ± 0.3	8.0 ± 3.7 (3.1–18)	37 ± 15 (5–67)	50.4 ± 20.3 (10–95)	Yes	Yes	–	Yes, only those who underwent concomitant procedures (3 d for miniphlebectomy ( *n* = 15), 7 d for sclerotherapy ( *n* = 19).
Koramaz et al 22	VariClose	150	0	0	3	–	6.88 ± 1.8 (5.5–15)	31.97 ± 6.83	7 (4–11)	No	No	No	No
	Evlas	189	0	0	0.5 a	N/A	7.15 ± 1.77 (5.5–14)	31.65 ± 6.25	18 (14–25)	No	No	No	Yes, class 2 × 2 wk.
Chan et al 26	VenaSeal	108	0	0	4	–	6.6 (2.3–11.4)	28 (15–41)	64 (28–116)	Yes	No	No	Yes, x 1 mo.
Bademci et al 31	VariClose	50	0	0	3	1.5 (1.3–2)	7 (5.5–9)	29.5 (25–36)	25 (20–36)	No	No	No	No
Yavuz et al 27	VenaBlock	538	0	0	4	0.87 ± 0.15 (0.4–1.39)	6.7 ± 1.7 (5.5–14.6)	25.7 ± 4.9 (10–43)	11.7 ± 4.9 (5–33)	No	No	No	No
Yang et al 21	VenaSeal	83	17	6	–	1.8 ± 0.1	–	43 ± 1	–	–	–	–	No
	Venefit	289	30	9	–	N/A	–	41 ± 1	–	–	–	–	–

Abbreviations: N/A, not applicable; SD, standard deviation.

Note: Gray, comparators; –, no information.

Evlas: Evlas Circular fiber EVLA kit (1,470 nm) (Biolas, Ankara, Turkey); Closurefast: Closurefast RFA catheter (VNUS Medical Technologies, San Jose, CA); Venefit: Venefit Targeted endovenous RFA therapy system (Medtronic of Canada Ltd, Vancouver, British Columbia).

a Distance to superficial epigastric vein.

#### *n* -Butyl-2-Cyanoacrylate

Mean age of the recruited population was 49.3 years and 64.8% were females. Most procedures from Turkey used the VariClose NBCA system (Biolas, FG Group, Ankara, Turkey). 10 19 20 22 29 30 31 One study used VenaBlock adhesive (Invamed, Ankara, Turkey). 27 The rest utilized the VenaSeal system (Medtronic, Dublin, Ireland). 12 13 21 25 26 28 32 33 34 All procedures commenced by cannulation of the target vein with an introducer needle under ultrasound guidance at the most distal point of reflux. The position of the delivery catheter tip distal to SFJ or SPJ ranged from 3 to 5 cm. The average volume of NBCA glue used was 1.3 mL (range 0.87–2 mL) to treat veins with a mean length of 30.8 cm (range 24–43 cm) and diameter of 7 mm (range 5.6–8 mm). Procedure technique varied depending on the choice of NBCA device.

For VenaSeal, two initial 0.09-mL glue aliquots were injected 1 cm apart, followed by 3 cm pullbacks between each trigger pull. Pressure with Ultrasound (US) probe was applied to occlude the SFJ/SPJ before dispensing the first two aliquots to prevent glue from entering the deep venous system. The first two injections were followed by 3 minutes of compression. US probe pressure was applied for 30 seconds after subsequent injections.

The VariClose system used a similar technique in terms of initial pressure to occlude the SFJ or SPJ before first injection. The trigger was pressed for 5 seconds while withdrawing the catheter by 10 cm (giving 0.06 mL of glue at 2 cm/s). Pressure over each 10-cm segment of treated vein was applied for 30 seconds. Once the entire vein was treated, a further 30 seconds of pressure over the entire target vein was applied. VenaBlock used a similar method.

Recording of duration of NBCA procedures was not standardized. Two prospective studies calculated duration from the time of insertion of NBCA delivery catheter to the time of withdrawal (mean 19.3 minutes). 28 33 The period from establishing venous access to applying the final bandages was taken as procedure time in another two prospective studies, with an average of 38.7 minutes. 13 32 An even broader timing interval extended from skin preparation to final bandaging, including phlebectomies (mean 64 minutes). 25 26 One operator performed the procedures under intravenous sedation, which further extended length of intervention. 13

#### Radiofrequency Ablation

Three studies compared NBCA with RFA. 12 19 21 The mean age of patients was 51 years and 72.8% were females. The devices used were ClosureFast (VNUS Medical Technologies, San Jose, CA) and Venefit (Medtronic of Canada Ltd, Vancouver, British Columbia, Canada). Both are similar and require perivenous tumescent anesthesia. Procedure duration was recorded in two RCTs and results were conflicting. 12 19 On one side, NBCA took longer than RFA (24 vs. 19 minutes, *p* < 0.01). 1 The other RCT identified a significant reduction in favor of NBCA (15.3 vs. 27.3 minutes, *p* < 0.001). 19 Neither documented the actual commencement and completion of recording.

#### Endovenous Laser Ablation

EVLA was performed on 246 females (50.8%). Mean age was 44.4 years. Evlas Circular fiber EVLA kit (Biolas, Ankara, Turkey) was used in all three studies. It operates at a wavelength of 1,470 nm and uses tumescent anesthesia. Peak temperature reaches 1200°C (compared with 120°C for RFA). One retrospective analysis mentioned the application of manual pressure over the treated vein during laser fiber withdrawal but its benefit in terms of promoting vein closure was not investigated. 22 Compression stockings were prescribed following all EVLA procedures and all agreed that EVLA took significantly longer than NBCA or RFA ( *p* < 0.001). 10 19 22

### Postoperative Success

#### Occlusion Rate

**Fig. 2:**
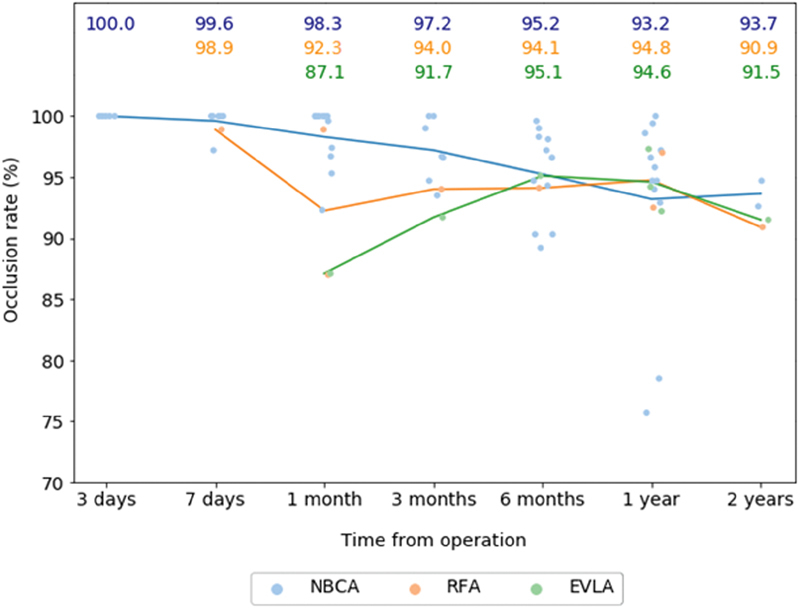
Categorical scatter point plot with the line of best fit representing the mean occlusion rates at each time interval. Color-coded numbers above the plots denote mean percentage occlusion rate.

Fig. 2 shows a substantial initial success rate after NBCA ablation followed by RFA and EVLA, respectively. Although limited, the 2-year NBCA data are superior. There is negligible difference between RFA and EVLA plots from 6 months onward. Partial and complete recanalization rates were lowest for NBCA throughout the period of follow-up.

#### Complications

**Fig. 3:**
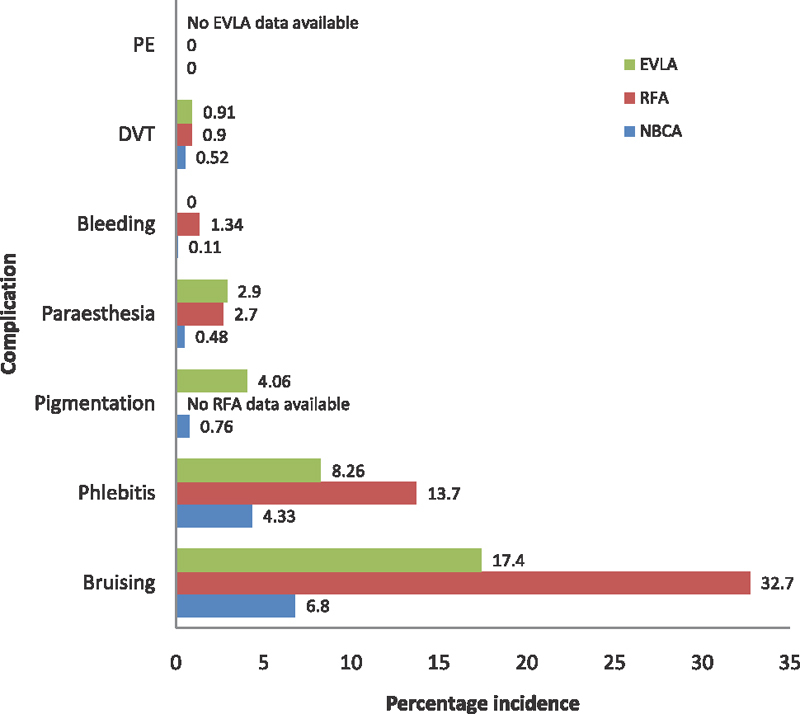
Bar chart displaying proportion of patients (%) experiencing a complication for each treatment modality. EVLA, endovenous laser ablation; NBCA, *n* -butyl cyanoacrylate; RFA, radiofrequency ablation.

There were no pulmonary embolic events. Nine cases of postablation DVT were observed in the NBCA group ( Fig. 3 ). 21 25 26 29 32 33 Four DVTs were reported in the RFA group and three following EVLA (endovenous heat-induced thrombi Class 1) without statistical significance. 12 19 21 22 All resolved with or without heparin treatment. Bruising was least in NBCA-treated patients. 1 25 26 All RCTs reported a statistically significant lower incidence of ecchymosis in the NBCA group. 1 10 19 One explanation is that repeated injections are required for tumescent anesthesia in thermal ablation methods while these are avoided in NBCA. 1 However, one retrospective comparative analysis found that five (2.65%) of EVLA-treated patients developed bruising which did not reach the level of significance compared with NBCA, even though such adverse event was absent in the latter cohort. 22 One prospective and one retrospective study by the same author using NBCA concluded that bruising resulted from stab avulsion sites which was performed in the same sitting. 25 26 Three studies documented minor point bruising at the access site of NBCA delivery catheter due to residual NBCA being applied close to the entry point. 27 31 33 Bleeding and hematoma formation were reported in one patient who underwent NBCA ablation and two post-RFA, the latter being at the site of vein access. 19 30 Paresthesia was temporary and less frequent in the NBCA group. 10 12 21 22 25 Seven patients complained of pigmentation at the treatment site after NBCA ablation which improved significantly over 1 year. 10 13 31 A higher number was reported after EVLA and were shown to be statistically significant. 22 All were temporary. Phlebitis after NBCA ablation was significantly less than post-RFA or EVLA. 21 22 One RCT reported the opposite, but failed to reach significance level. 1 Most reactions were transient and self-limiting or resolved with a short course of nonsteroidal anti-inflammatory drugs. 1 13 26 32 33 Antibiotics were prescribed in two studies. 22 29 Supplementary Fig. 2 compares the different NBCA glue products with the proportion of veins having postoperative phlebitis. Although inconsistently and heterogeneously recorded, intraoperative pain experience was least for cyanoacrylate procedures, presumably because of the lack of tumescent anesthesia and heat generation. It was therefore better tolerated. 10 19 32 Most subjects returned to work the following day and this was superior to RFA and EVLA. 19 20 25 26 32 One patient developed generalized urticaria after the first week of treatment indicating delayed NBCA allergy. This settled with oral antihistamines and steroids. 32

### VCSS and Quality of Life Scores

All endovenous ablation modalities exhibited a statistically significant decline in VCSS scores over time. 10 12 19 20 22 25 26 27 28 29 31 33 34 Two RCTs reported no difference between NBCA and EVLA during follow-up and another favored NBCA at 2 years ( *p* < 0.001). 10 19 22 Two prospective analyses by Gibson and Park were analyzed separately because they used the revised version of VCSS. 35 Mean baseline scores were 6.5 ± 2.4 (3–14) and 4.3 ± 2.1 (2–13). At 30 days, these improved respectively to 1.8 ± 1.4 (0–6) and 1.2 ± 1.0 (0–5) ( *p* < 0.001 and 0.024). 13 32

The AVVQ was the main reporting modality for quality of life. Its downward decline from baseline was significant, consistent, and similar in all groups. Few manuscripts utilized other quality of life scores including EQ-5D, EQ-5D TTO, CIVIQ, and SF-36. All except SF-36 exhibited a significant improvement from baseline. 1 12 25 26 29 32 33 34

### Influence of Variables on Occlusion Rate

Occlusion rate after cyanoacrylate glue treatment is not influenced by vein length, diameter, dispensing device, or use of postoperative compression stockings ( Table 4 ).

**Table 4:** Analysis of the effect of four variables on occlusion rate of NBCA-treated veins (Spearman's correlation, Mann-Whitney U test ^b^ )

	*p* -Values			
Occlusion rate interval	Vein length ^a^	Vein diameter ^a^	NBCA device ^b^	Compression stockings ^b^
3 mo	0.728	0.538	0.593	0.564
6 mo	0.423	0.413	0.295	0.521
12 mo	0.931	0.160	0.873	0.240

Abbreviation: NBCA, *N* -butyl-2-cyanoacrylate.

## Discussion

Monomeric cyanoacrylate compounds polymerize upon contact with anionic components of plasma, a process consisting of three distinct phases: initial rapid polymerization with linear increase in tensile forces lasting approximately 10 seconds (phase 1), stable tensile forces lasting approximately 60 seconds (phase 2) followed by a more rapid rise of tensile forces (phase 3). 36 The process of luminal fibrosis after glue injection takes several weeks before it becomes permanent. 37 Adjunctive treatments (phlebectomy or foam sclerotherapy) risk a type 2 error and the confounding potential of these treatments is a subject of future trials. 10 12 13 19 20 25 26 27 29 33

There were outliers that skewed the NBCA occlusion data at 6 months and 1 year, leading to a dip in success rate at these intervals. 25 26 30 Bissacco et al reviewed 1,000 NBCA cases in seven studies (two prospective, four retrospective) and found 96.8% of veins occluded at 12 months. 38 Two studies reported NBCA occlusion beyond the 2-year interval, and these were 94.1% at 30 months and 94.7% at 36 months, respectively. 20 28 Time to complete occlusion was shorter for NBCA than any of the endothermal modalities because veins are instantly occluded by approximation of their intima, while thermal ablation is dependent on vein wall destruction and subsequent fibrosis—a biological process which takes longer. 12 The outcomes of RFA versus EVLA have been extensively studied in previous trials. Using the ClosureFast RFA system on 200 limbs (163 GSVs and 41 SSVs), Choi et al reported 94.6% occlusion in GSV and 94.5% in SSV at 13.9 months, which is similar to our data. 39 A prospective double-blind RCT comparing RFA versus EVLA (159 patients—79 RFA, 80 EVLA) by Nordon et al identified a 100% occlusion at 7 days. The 3-month occlusion rate reached 97% for RFA and 96% for EVLA. There was no significant difference between the groups. 40 In the LARA study, Goode et al reported 95 and 74% occlusion rate for RFA at 10 days and 9 months respectively. For EVLA, these were 95 and 78%. The high failures at 9 months were attributed to incorrect setting on the RFA which improved to 98% upon adjustment. No reasons for EVLA failures were given but the short wavelength of the laser used (810 nm) and pullback speed might be implicated. 41

Recanalization does not necessarily signify return of symptoms as many maintain a good quality of life and anticoagulation does not appear to be a predisposing factor. 12 25 27 29 NBCA is noninferior to RFA in terms of freedom from recanalization. 12 Chan et al found a significant risk in their earlier study with vein diameters ≥8 mm, which was reduced to ≥6.6 mm in a subsequent analysis. 25 26 This was contradicted in the WAVES study which reported 100% occlusion at 30 days using the same NBCA system. However, the latter allowed operators to inject additional glue in larger veins according to their discretion. 32 Other reported determinants of failure were operator experience, anatomical variation (e.g., aneurysms, junction of large varicosities), 29 development of incompetency in a once competent vein, intraluminal thrombus formation (most relevant for failure after thermal ablation), and missing the vein altogether. 12 22 25 26 29

There is no officially reported incidence of DVT for NBCA but it is understood to be very low especially if tip of catheter is positioned 5 cm away from the superficial-to-deep vein junction. RFA carries a risk of 0 to 16% and EVLA 0 to 7.7%. Routine postoperative DUS may pick up asymptomatic thrombi. 39 The benefit of anticoagulation for such DVTs is debatable as most resolve spontaneously. Ultrasound guidelines distinguishing thrombus from glue are also lacking. 26 No details about length of stockings were provided (example: thigh high or below knee). Bruising was least after NBCA, particularly when glue injection was stopped 2 cm proximal to the catheter entry site. 27 A modern laser with longer wavelength (1,470 nm) causes less ecchymosis than one with shorter wavelength (810 nm) because it is less damaging to the vessel wall. 2 41 Prior to this improvement in laser technology RFA was deemed superior to EVLA with regards to postprocedural bruising. 19 40 41 Other factors implicated in ecchymosis include the use of tumescent anesthesia, phlebectomies, anticoagulants, body mass index, and ethnicity. 40 Paresthesia typically occurs in 1 to 2% of cases post-RFA and EVLA, and is rare after NBCA. In the latter it is often mild and self-limiting. 2 A few recent studies and case reports address the issue of hypersensitivity reactions causing phlebitis-like signs and symptoms in veins treated with cyanoacrylate glue. Generally these respond well to antihistamines and/or steroids, and may even resolve spontaneously. In those veins requiring excision, histological examination identified features of a type IV hypersensitivity reaction to the glue (foreign body). 42 43 44 This is different from the phlebitis encountered after thermal ablation. Patients should be asked about cyanoacrylate allergy preoperatively to minimize risks. 45

This systematic review has some limitations. The comprehensive literature search and data extraction were performed by one author. It excluded mechanochemical ablation and the period of follow-up was short. A meta-analysis would have been ideal but as highlighted in a recent article, the scarcity and heterogeneity of RCTs made this difficult. 38 As most patients were not sedated, double blinding was impossible. Outcome assessors were often the same ones recruiting, carrying out the treatments and/or following-up patients. This was taken into consideration in part by modifying the Cochrane risk of bias tool. 15 Some methodologies opted for an induction period to cater for the “learning curve” but others did not. 12 34 One major inconsistency was in the duration of procedures. There are no set standards as to when time-keeping should start and stop. The lack of reproducibility makes these measurements unreliable.

In terms of patient characteristics, one study included more smokers in the NBCA group and another deviated its protocol to include a patient with higher BMI. 12 28 No differentiation between unilateral or bilateral treatment of varicose veins was made. 25 26 “Return to normal activities” needs better definition, as these activities are different in an elderly or morbidly obese patient compared with a healthy fit subject. Reflux is best detected in the standing posture on DUS as recommended by the European Society for Vascular Surgery (2015), but some measured this supine. 2 Lastly, it would be interesting to see a trial addressing NBCA use for varicose veins in anticoagulated patients.

## Conclusion

This systematic review shows the potential benefits of cyanoacrylate glue over RFA and EVLA. Due to its immediate action, occlusion is retained even without postoperative elastic bandages or compression stockings. Patients experienced less pain as there was no tumescent anesthesia, multiple injection sites, or heat involved. Phlebitis is often mild, self-limiting, and attributed to localized skin reaction to the glue. It can be managed conservatively. Procedure times are generally short and patients typically resume work on day 1 or 2. Failure rates are less but longer-term data are required to affirm this. Cyanoacrylate ablation carries less risk of paresthesia, ecchymosis, and eliminates burn injuries. The two most readily available NBCA kits can be used on various lengths and diameters of veins (including bilateral cases of appropriate length with a single vial of glue). 25
