# Supplementary material for: Efficacy of Cyanoacrylate Glue Ablation of Primary Truncal Varicose Veins Compared to Existing Endovenous Techniques: A Systematic Review of the Literature
Source: Surg J (N Y). 2020 Jun 19;6(2):e77–86. doi: 10.1055/s-0040-1708866 (PMC7305022; doi:10.1055/s-0040-1708866)
Supplement: Supplementary file 1 — Supplementary Material [file 10-1055-s-0040-1708866-s1900086oa.pdf]

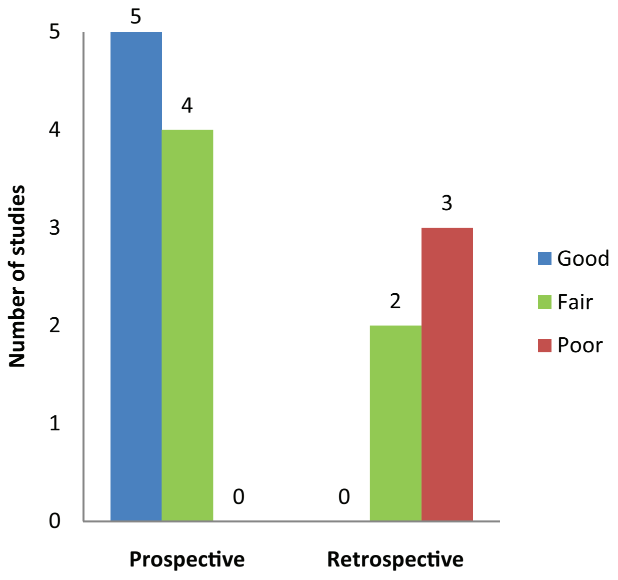

Supplementary Fig. 1 Quality rating of noncomparative studies reported using the National Heart, Lung and Blood Institute: quality assessment tool for before–after (pre–post) studies with no control group.

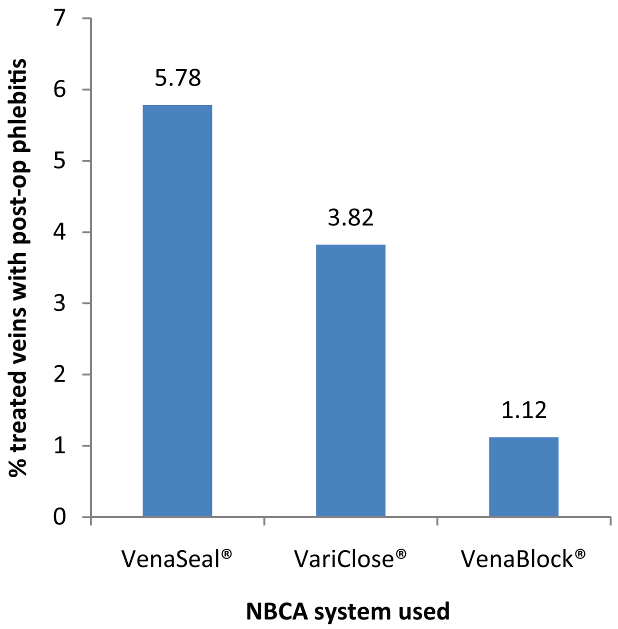

Supplementary Fig. 2 Bar chart showing the percentage incidence of postoperative phlebitis in cyanoacrylate-treated veins for each of the three NBCA-dispensing devices used. NBCA, *n*-butyl-2-cyanoacrylate.

Supplementary Table 1 Patient factors for inclusion and exclusion[1,2,10,11]

Inclusions	Exclusions
≥18 y of age<br>DUS postintervention<br>SFJ or SPJ incompetence with reflux >0.5 s on DUS<br>CEAP class: C1–C6<br>Dilated GSV or SSV on clinical examination (with or without symptoms)	Pregnancy<br>Breastfeeding<br>Recurrent varicose veins<br>Secondary varicose veins<br>Nontruncal varicosities<br>History of DVT or PE

Abbreviations: CEAP, clinical, etiological, anatomical and pathophysiological classification of chronic venous disease; DUS, duplex ultrasound scan; DVT, deep vein thrombosis; GSV, great saphenous vein; PE, pulmonary embolism; SFJ, saphenofemoral junction; SPJ, saphenopopliteal junction; SSV, small saphenous vein.

Supplementary Table 2 Quality rating: good, fair, or poor

	Was the study question or objective clearly stated?	Were eligibility/selection criteria for the study population prespecified and clearly described?	Were the participants in the study representative of those who would be eligible for the test/service/intervention in the general or clinical population of interest?	Were all eligible participants that met the prespecified entry criteria enrolled?	Was the sample size sufficiently large to provide confidence in the findings?	Was the test/service /intervention clearly described and delivered consistently across the study population?	Were the outcome measures prespecified, clearly defined, valid, reliable, and assessed consistently across all study participants?	Were the people assessing the outcomes blinded to the participants' exposures/interventions?	Was the loss to follow-up after baseline 20% or less? Were those lost to follow-up accounted for in the analysis?	Did the statistical methods examine changes in outcome measures from before to after the intervention? Were statistical tests done that provided *p* values for the pre-to-post changes?	Were outcome measures of interest taken multiple times before the intervention and multiple times after the intervention (i.e., did they use an interrupted time-series design)?	If the intervention was conducted at a group level (e.g., a whole hospital, a community, etc.) did the statistical analysis take into account the use of individual-level data to determine effects at the group level?	Overall quality rating
Park[13]	Yes	Yes	Yes	Yes	CD	No	Yes	No	No	Yes	No	NA	Fair
Eroglu[20]	Yes	Yes	Yes	Yes	CD	Yes	Yes	No	Yes	Yes	Yes	NA	Good
Almeida[28]	Yes	Yes	CD	Yes	CD	Yes	Yes	No	No	Yes	Yes	NA	Fair
Kolluri[34]	Yes	Yes	Yes	Yes	CD	Yes	Yes	No	Yes	Yes	Yes	NA	Good
Gibson[32]	Yes	Yes	Yes	Yes	CD	Yes	Yes	No	Yes	Yes	Yes	NA	Good
Tekin[30]	Yes	Yes	Yes	Yes	CD	Yes	Yes	NR	Yes	No	Yes	NA	Fair
Çalik[29]	Yes	Yes	Yes	Yes	CD	Yes	Yes	No	Yes	Yes	Yes	NA	Good
Chan[25]	Yes	Yes	Yes	CD	NR	Yes	Yes	No	Yes	Yes	Yes	NA	Fair
Proebstle[33]	Yes	Yes	Yes	Yes	CD	Yes	Yes	No	Yes	Yes	Yes	NA	Good
Yavuz[27]	Yes	Yes	Yes	CD	CD	No	Yes	NA	Yes	Yes	Yes	NA	Poor
Yang[21]	Yes	NR	CD	NR	CD	Yes	Yes	No	Yes	Yes	No	NA	Poor
Chan[26]	Yes	Yes	Yes	CD	CD	Yes	Yes	No	Yes	Yes	Yes	NA	Fair
Bademci[31]	No	Yes	Yes	CD	CD	Yes	No	NR	Yes	Yes	Yes	NA	Poor
Koramaz[22]	Yes	Yes	Yes	CD	CD	Yes	Yes	No	Yes	Yes	No	NA	Fair

Abbreviations: gray, prospective cohort studies; blue, retrospective analyses; CD, cannot determine; NA, not applicable; NR, not reported.
